# Johannes Stark und die gescheiterte Erklärung deutscher Nobelpreisträger zur Volksabstimmung vom 19. August 1934

**DOI:** 10.1002/bewi.70004

**Published:** 2025-10-27

**Authors:** Dieter Hoffmann, Andreas Kleinert

**Affiliations:** ^1^ Abteilung Molekülphysik Fritz‐Haber‐Institut der Max‐Planck‐Gesellschaft Faradayweg 4-6 D‐14195 Berlin Germany; ^2^ Zentralmagazin Naturwissenschaftlicher Sammlungen Martin‐Luther‐Universität Halle‐Wittenberg Domplatz 4 D‐06108 Halle Germany

**Keywords:** Nobelpreisträger (deutsche), Volksabstimmung 1934, Wissenschaft im Nationalsozialismus, Heisenberg, W., Laue, M.v., Nernst, W., Planck, M., Stark, J.

## Abstract

Drawing on previously unknown sources, this article documents the physicist Johannes Stark's unsuccessful attempt to publish a declaration by German Nobel laureates in support of the August 1934 referendum. Following the death of Reich President Paul von Hindenburg, this referendum aimed to legitimize the transfer of the two highest state offices—Reich President and Reich Chancellor—to one single person, thereby making Adolf Hitler the head of state and Führer of the German people. At the suggestion of the Propaganda Ministry, Stark sent a telegram to eleven “Aryan German Nobel laureates” in chemistry and physics, and urged them to sign a public appeal to participate in the referendum and support “the great national commitment of the entire German people” to Adolf Hitler as “Führer of the German people.” Responses to Stark's telegram ranged from wholehearted support to rejection. The majority of the laureates declined to lend their signature, and the initiative ultimately failed. The episode reveals that Stark—although at the height of his influence as president of the Physikalisch‐Technische Reichsanstalt (Imperial Institute of Physics and Technology) and the Deutsche Forschungsgemeinschaft (German Research Foundation)—was largely isolated within the scientific community of the so‐called Third Reich.

Johannes Stark (1874–1957) hatte zu Beginn des zwanzigsten Jahrhunderts mit brillanten Experimenten gezeigt, dass er zu den herausragenden Physikern seiner Zeit gehörte.^1^ Für den Nachweis der Aufspaltung der Spektrallinien im elektrischen Feld (Stark‐Effekt), der ihm nach jahrelangen Vorarbeiten 1913 gelungen war, wurde er im Jahre 1919 mit dem Nobelpreis für Physik geehrt. Darüber hinaus gehörte er zu den frühen Pionieren der Quantentheorie und wurde so ein Repräsentant der physikalischen Moderne.^2^ Allerdings galt er bei seinen Zeitgenossen auch als Einzelgänger und Querulant. Die politischen Zeitumstände, namentlich der Erste Weltkrieg mit der deutschen Niederlage und dem Untergang des deutschen Kaiserreichs, die zur Konstituierung der Weimarer Republik führte, verstärkten nicht nur seine konservative und nationalistische Weltsicht, sondern machten ihn in gesellschaftlichen und wissenschaftspolitischen Fragen zum Außenseiter, wobei er von sich selbst in einem Brief von 1915 als „chauvinistischer Physiker“ sprach.^3^ In der Weimarer Republik erfolgte überdies eine politische Radikalisierung seiner Außenseiterrolle, die ihn zu einem glühenden Verehrer Adolf Hitlers und nachfolgend (1930) auch zum Parteigänger der Nationalsozialisten machte.^4^ Seine politische Radikalisierung ging einher mit einer zunehmenden Kritik der modernen Physik, namentlich von Quantenphysik und Allgemeiner Relativitätstheorie und ihrer führenden deutschen Repräsentanten Albert Einstein, Walther Nernst, Max Planck und später auch Werner Heisenberg, die für ihn Vertreter des „jüdischen Geistes“ in der Wissenschaft, speziell in der modernen Physik waren. In einem Beitrag der SS‐Zeitschrift *Das Schwarze Korps* stellte er sogar absichtsvoll einen Bezug zwischen Heisenberg und Carl von Ossietzky her, dem Nazi‐Gegner und linken Pazifisten, der seit 1933 in KZ‐Haft war und dessen Auszeichnung mit dem Friedens‐Nobelpreis 1936 von Hitler mit der Anordnung erwidert wurde, deutschen Wissenschaftlern die Annahme des Nobelpreis generell zu verbieten; auch prägte er in diesem Zusammenhang den denunziatorisch‐diffamierenden Begriff von den „weißen Juden in der Wissenschaft“.^5^

Starks Status als „alter Kämpfer“ der nationalsozialistischen Bewegung half 1933, ein zehnjähriges Interregnum als Privatgelehrter zu beenden. Querelen mit seinen Kollegen an der Würzburger Universität hatten im Frühjahr 1922 dazu geführt, dass er kurzerhand sein Physik‐Ordinariat niederlegte und sich als Privatgelehrter und Unternehmer in seine oberpfälzische Heimat zurückzog. Allerdings konnte das Starks Ehrgeiz auf Dauer nicht befriedigen, so dass er ab Mitte der zwanziger Jahre wieder versuchte, in der akademischen Welt Fuß zu fassen. Seine Bemühungen blieben indes ohne Erfolg, wozu sicherlich seine aggressive Kritik der modernen Physik und des „schwarz‐roten Systems“ der Weimarer Republik beigetragen hat, so dass er trotz des Nobelpreisträger‐Nimbus in der damaligen scientific community weitgehend isoliert war.

Als Adolf Hitler am 30. Januar 1933 die politische Macht in Deutschland übertragen wurde, schrieb Stark euphorisch an seinen Physikerkollegen und politischen Gesinnungsgenossen Philipp Lenard:

Endlich ist die Zeit gekommen, da wir unsere Auffassung von Wissenschaft und Forschern zur Geltung bringen können. Ich habe gleich die Gelegenheit meines Glückwunschschreibens an Minister Frick, mit dem ich persönlich bekannt bin, benützt, ihn darauf hinzuweisen, dass Sie und ich ihm bei der Einflussnahme auf die ihm unterstellten wissenschaftlichen Institute gern unseren Rat zur Verfügung stellen werden.^6^

Starks Bemühungen waren nun von Erfolg gekrönt – Wilhelm Frick, Innenminister im Kabinett Hitler, ernannte ihn zum 1. Mai 1933 zum Präsidenten der Physikalisch‐Technischen Reichsanstalt (PTR) in Berlin. Neben den Instituten der Kaiser‐Wilhelm‐Gesellschaft war die PTR die bedeutendste außeruniversitäre Forschungseinrichtung im Bereich der Naturwissenschaft – speziell der Physik – in Deutschland, mit einem hohen internationalen Renommee.^7^

Auch wenn Starks Ernennung „gegen das einhellige Votum aller befragten Fachmänner“ geschah – wie Max von Laue nach dem Krieg berichtete^8^ –, war er damit nicht nur ins akademische Leben zurückgekehrt, sondern es war auch der Grundstein für seinen Aufstieg zu einem der bekanntesten, einflussreichsten und mächtigsten Gelehrten in der Frühzeit des Dritten Reichs gelegt. So folgte im folgenden Jahr die Übernahme des prestigeträchtigen und mächtigen Präsidentenamtes der Deutschen Forschungsgemeinschaft (DFG). Damit hatte Stark Schlüsselpositionen der nationalsozialistischen Wissenschafts‐ und Forschungspolitik übernommen. Allerdings verblich sein Stern relativ schnell: Bereits 1936 hatte man ihn im Ergebnis einer machtpolitischen Intrige aus dem Amt des DFG‐Präsidenten gedrängt^9^ und 1939 wurde er als PTR‐Präsident in den altersbedingten Ruhestand versetzt.^10^ Mit Fanatikern und Ideologen wie Stark und anderen Anhängern der sogenannten „Deutschen Physik“ ließ sich zwar die „nationale Revolution“ vorantreiben, doch für den autarken Wehrstaat, der seit Mitte der dreißiger Jahre für die expansionistischen Ziele des Dritten Reiches errichtet wurde, waren moderne Physik und Technik, die im Dissens zur „Deutschen Physik“ standen, prioritär.^11^

Mit seinen Meriten war Stark für die nationalsozialistische Propaganda attraktiv, so für die Propagandakampagne, die nach dem Tod von Reichspräsident Paul von Hindenburg im Sommer 1934 in Szene gesetzt wurde. Mit dem Tod Hindenburgs^12^ bot sich für Adolf Hitler die Gelegenheit, durch Zusammenlegung der beiden höchsten Staatsämter, das des Reichskanzlers und das des Reichspräsidenten, seine autokratischen Machtfülle weiter auszubauen und sich als „Führer des deutschen Volkes“ zu inthronisieren. Als Führer und Reichskanzler sollte er künftig auch über die Ernennung und Abberufung der Reichsminister sowie über die Auflösung des Reichstags entscheiden, was bisher dem Reichspräsidenten vorbehalten war. Vor allem aber würde Hitler mit der Zusammenlegung der Ämter auch zum Oberbefehlshaber von Heer, Marine und Luftwaffe werden, deren Vereidigung fortan auf ihn erfolgen sollte.

Diese Machtkonzentration war von langer Hand geplant – u.a. durch das Gesetz über Volksabstimmungen vom 14. Juli 1933, das die Möglichkeit eröffnete, an den gesetzgebenden Körperschaften vorbei Gesetze und andere Maßnahmen der Reichsregierung zu verabschieden. So war angesichts des zu erwartenden Todes von Hindenburg bereits am 1. August 1934 vom Kabinett Hitler ein Gesetz über das Staatsoberhaupt des Deutschen Reiches erlassen worden, das im Falle des Todes des Reichspräsidenten, der bereits am folgenden Tag (2. August 1934) eintrat, die Vereinigung der beiden höchsten Staatsämter legitimierte. Allerdings sollte die Ernennung zum Führer und Reichskanzler auf Anordnung Hitlers „die ausdrückliche Sanktion des deutschen Volkes“ erhalten, d.h. das entsprechende Gesetz bzw. der Beschluss des Kabinetts war „unverzüglich dem deutschen Volke zur freien Volksabstimmung vorzulegen“.^13^ Noch am Todestag von Hindenburg legte eine Regierungsverordnung den 19. August 1934 als Tag der Volksabstimmung fest und eine Durchführungsverordnung des Reichs‐Innenministers vom folgenden Tag regelte die technischen Details der Volksabstimmung und die Form des Stimmzettels mit der Abstimmungsfrage. Gefragt wurde: “Stimmst Du, deutscher Mann, und Du, deutsche Frau, der in diesem Gesetz [über das Staatsoberhaupt des Deutschen Reiches] getroffenen Regelung zu?”^14^

Die zügige Übereinkunft für den Volksentscheid und die schnelle Verabschiedung der dazu nötigen Regierungsbeschlüsse zeigen, dass dieser gründlich und bis in Details vorbereitet war. Dies wird dadurch unterstrichen, dass sofort eine von Goebbels inszenierte und groß angelegte Agitation in Gang gesetzt wurde, die alles bis dahin Gekannte in den Schatten stellte – „Goebbels hat sich selbst überboten“, kommentierte sarkastisch der SPD‐Vorstand im Prager Exil die Situation.^15^ In der gleichgeschalteten Presse erschienen in den verbleibenden gut zwei Wochen bis zum Volksentscheid täglich umfangreiche Berichte und Zustimmungserklärungen. Dabei kamen nicht nur persönliche Bekenntnisse von begeisterten Volksgenossen zum Abdruck, sondern auch Akklamationen von Berufsverbänden oder Berufsgruppen; namentlich bekannten sich – wie schon bei der „Schicksalswahl“ vom März 1933^16^ – auch die „Vertreter der Deutschen Wissenschaft“ zur „Führerschaft Adolf Hitlers, dass er das Deutsche Volk aus seiner Not und Bedrückung herausführen wird.“^17^

In diese Propaganda‐Kampagne ordnet sich die Initiative Johannes Starks ein, der am 11. August 1934 telegrafisch die „arischen deutschen Nobelpreisträger“ aufforderte, in einer öffentlichen Stellungnahme zur Teilnahme am Volksentscheid aufzurufen und sich zu Adolf Hitler als dem „Retter und Führer des deutschen Volkes“ zu bekennen. Dass sich Stark zum Initiator eines solchen Bekenntnisses für den Nationalsozialismus und namentlich für Adolf Hitler machte, überrascht nicht, gehörte er doch nicht nur zum elitären Kreis der Nobelpreisträger – der Nobelpreis galt damals (wie auch heutzutage) unter Wissenschaftlern und hinsichtlich seiner gesellschaftlichen Reputation als die höchste Auszeichnung, mit der ein Gelehrter geehrt werden kann^18^ –, sondern er zählte, wie bereits erwähnt, ebenfalls seit Mitte der zwanziger Jahre zu den frühen und glühenden Verehrern Adolf Hitlers und seiner Bewegung.

Die Initiative für eine Aktion deutscher Nobelpreisträger zum anstehenden Volksentscheid ging jedoch nicht von Johannes Stark selbst aus, sondern war durch das Goebbelssche Propagandaministerium an ihn herangetragen worden. Ob die Anregung von Joseph Goebbels selbst ausging oder eine Idee der Ministerialbürokratie und speziell des Oberregierungsrats Wilhelm Ziegler war, ist nicht klar – in einem Brief Starks an Propagandaminister Joseph Goebbels vom 23. August, also vier Tage nach dem Referendum, findet man nur den Hinweis: „Auf Veranlassung Ihres Herrn Oberregierungsrates Dr. Ziegler habe ich gerne den Versuch gemacht, eine öffentliche Kundgebung der 12 arischen deutschen Nobelpreisträger für Adolf Hitler herbeizuführen.“^19^ Wilhelm Ziegler (1891–1962) war 1925 an der Universität Frankfurt mit einer historischen Arbeit promoviert worden; in seiner Stammkarte ist als besonderes Forschungsgebiet „Neuere Geschichte u. Judenfrage“ ausgewiesen.^20^ Mit Gründung des Reichsministeriums für Volksaufklärung und Propaganda im März 1933 war er dort Referent für Wissenschaft geworden und gehörte zum engeren Kreis um den Minister, der die Propagandakampagne zum Volksentscheid steuerte. Ob sich Stark und Ziegler bereits vor 1934 gekannt haben, ist ungewiss, doch blieb der Kontakt danach bestehen – so haben sich Korrespondenzen anlässlich des 50jährigen Jubiläums der PTR im Jahre 1937 erhalten, bei denen Stark mit Hilfe Zieglers versuchte, Goebbels für die Schlussansprache auf dem Festakt der Reichsanstalt im November 1937 zu gewinnen, der zudem „das ‚Sieg‐Heil‘ auf den Führer ausbringen“ sollte.^21^ Im folgenden Jahr wurde Ziegler nochmals von Stark hinsichtlich eines geplanten Aufsatzes für die englische Zeitschrift *Nature* konsultiert und gefragt, ob dieser „den Grundsätzen der deutschen Auslandspropaganda auf kulturellem Gebiet entspricht“.^22^

Die Idee, für das anstehende Plebiszit eine Kundgebung der „arischen“ deutschen Nobelpreisträger zu organisieren, muss unmittelbar nach Verabschiedung der Kabinettsbeschlüsse und dem Start der Goebbelsschen Propagandakampagne, d.h. in der ersten Augustwoche 1934 entstanden sein. Somit war Eile geboten, weshalb Stark bereits in der Folgewoche, am 11. August 1934, das oben erwähnte Telegramm versandte. Es hatte den Wortlaut:

Umgehende Antwort erbeten, ob Sie zusammen mit anderen Nobelpreisträgern folgende Kundgebung unterzeichnen wollen:

In Adolf Hitler verehren und bewundern wir deutsche Naturforscher den Retter und Führer des deutschen Volkes; unter seinem Schutz und seiner Förderung wird unsere wissenschaftliche Arbeit dem deutschen Volke dienen und das deutsche Ansehen in der Welt mehren.

gez. Stark. Notgemeinschaft der deutschen Wissenschaft, Schloß, Portal II.^23^

Adressaten waren die folgenden Nobelpreisträger (in alphabetischer Ordnung mit Angabe des Fachgebiets und dem Jahr der Ehrung):

Friedrich Bergius (Chemie 1931)

Carl Bosch (Chemie 1931)

Hans Fischer (Chemie 1930)

Werner Heisenberg (Physik 1932)

Gustav Hertz (Physik 1925)

Max von Laue (Physik 1914)

Philipp Lenard (Physik 1905)

Walther Nernst (Chemie 1920)

Max Planck (Physik 1918)

Heinrich Otto Wieland (Chemie 1927)

Adolf Windaus (Chemie 1928)

Die Zusammenstellung zeigt, dass Stark nicht *die* „arischen“ deutschen Nobelpreisträger für eine gemeinsame Kundgebung einlud, sondern allein die aus den ihm nahestehenden Fachdisziplinen Chemie und Physik. Unberücksichtigt blieben damit die Laureaten der Sparte Medizin mit den Preisträgern Otto Meyerhoff (1922) und Otto Warburg (1931). Für ihren Ausschluss war sicherlich die „nichtarische“ Herkunft maßgebend: Meyerhoff, den Stark in einem Brief an Lenard als „Münchener Chemiejuden“ geschmäht hatte,^24^ war Kind jüdischer Eltern, und Otto Warburg hatte in der väterlichen Linie zwei jüdische Großeltern, womit er ein sogenannter „Halbjude“ war. Ihre Forschungen und speziell ihre preisgekrönten Arbeiten waren indes stark biochemisch orientiert und hatten nur wenig mit klassischer medizinischer Forschung gemein, womit sie durchaus das Starksche Kriterium eines „Naturforschers“ erfüllt hätten. Beide waren zudem im Sommer 1934 als Direktoren von Kaiser‐Wilhelm‐Instituten in Heidelberg bzw. Berlin noch in Amt und Würden. Meyerhoff wurde erst 1938 aus seinem Amt gedrängt und emigrierte über die Schweiz und Frankreich in die USA.^25^ Warburg konnte sich über die Zeit des Dritten Reichs hinweg als KWI‐Direktor halten;^26^ nicht zuletzt, weil er 1942 erfolgreich einen Antrag auf „Arisierung“ gestellt hatte, womit er „Deutschblütigen“ gleichgestellt wurde.^27^

Die beiden anderen Nobelpreiskategorien, Literatur und Frieden, haben Stark denkbar ferngestanden und sind wohl deshalb unberücksichtigt geblieben. Ohnehin gab es damals in diesen Sparten nur drei deutsche Preisträger: Gerhart Hauptmann (Literatur 1912), Thomas Mann (Literatur 1929) und Ludwig Quidde (Frieden 1927). Alle drei waren „Arier“, jedoch prominente Kritiker des Nationalsozialismus und speziell Hitlers. Quidde war zudem bekennender Pazifist und lebte seit März 1933 im Schweizer Exil, das zunächst auch Mann gewählt hatte, bevor er 1938 in die USA ging. Was Hauptmann betrifft, so war sein Verhältnis zum Dritten Reich keineswegs so eindeutig wie bei seinem Schriftstellerkollegen Thomas Mann; beispielsweise hatte er 1933 eine Loyalitätserklärung der Preußischen Akademie der Künste für Hitler unterzeichnet,^28^ die für Mann hingegen Grund war, seinen Austritt aus der Akademie zu erklären.^29^

Auch von den damals lebenden deutschen Nobelpreisträgern für Physik und Chemie – insgesamt sind es 15 (in Tab. 1 fett ausgezeichnet) – wurden nicht alle mit Starks obigem Telegramm bedacht. In den Findbüchern zu den Nachlässen von Albert Einstein,^30^ James Franck^31^ und Richard Willstätter^32^ finden sich keinerlei Spuren einer entsprechenden Aufforderung oder gar Korrespondenz mit Stark. Sie waren als Adressaten außen vor geblieben – wahlweise wegen ihrer „nichtarischen“ Herkunft, der öffentlichen Ablehnung der nationalsozialistischen Gewaltherrschaft oder weil sie emigriert waren.

**Tab. 1:** Die deutschen Nobelpreisträger 1901–1933 (die zum Zeitpunkt der Volksabstimmung im August 1934 noch lebenden sind fett ausgezeichnet, die von J. Stark angeschriebenen zudem durch eine Unterstreichung markiert.)

	**Physik**	**Chemie**	**Medizin**	**Literatur**	**Frieden**
**1901**	W. C. Röntgen		E. v. Behring		
**1902**		E. Fischer		Th. Mommsen	
					
					
**1905**	**Ph. Lenard**	A. v. Baeyer	R. Koch		
					
		E. Buchner			
**1908**			P. Ehrlich	R. Eucken	
**1909**	F. Braun	W. Ostwald			
**1910**		O. Wallach	W. Kossel	Heyse	
**1911**	W. Wien				
				**G. Hauptmann**	
					
**1914**	**M. v. Laue**				
**1915**		**R. Willstätter**			
					
					
**1918**	**M. Planck**	F. Haber			
**1919**	**J. Stark**			**Th. Mann**	
**1920**		**W. Nernst**			
**1921**	**A. Einstein**				
**1922**			**O. Meyerhoff**		
					
					
**1925**	**J. Franck** **G. Hertz**				
					G. Stresemann
**1927**		**H.O. Wieland**			**L. Quidde**
**1928**		**A. Windaus**			
					
**1930**		**H. Fischer**			
**1931**		**F. Bergius** **C. Bosch**	**O. Warburg**		
**1932**	**W. Heisenberg**				
**1933**	**E. Schrödinger**				

Dazu gehört auch der Physiker Erwin Schrödinger, der gleichzeitig mit Heisenberg im Dezember 1933 den Preis erhalten hatte und eigentlich Österreicher war. Als er 1927 zum Nachfolger von Max Planck als Professor für theoretische Physik der Berliner Universität berufen wurde, musste er infolge des deutschen Beamtenrechts die deutsche Staatsbürgerschaft annehmen. Obwohl er „Arier“ war und ihm auch keine politische Verfolgung im nationalsozialistischen Deutschland drohte, hatte er sich im Herbst 1933 von der Berliner Universität beurlauben lassen und demonstrativ in Oxford eine Gastprofessur übernommen.^33^ Damit zählte er für Stark zu den verpönten Emigranten; auch stand Stark der Schrödingerschen Wellenmechanik höchst kritisch gegenüber, die für ihn „undurchsichtig und formalistisch“^34^ war und dessen Schöpfer „sich in seinen Theorien vom jüdischen Geist hat anstecken lassen“.^35^ In diesem Sinne wurde auch Schrödingers spätere Ehrung mit der prestigeträchtigen Planck‐Medaille der Deutschen Physikalischen Gesellschaft (1937) im SS‐Organ *Das Schwarze Korps* als „eine nationale Entwürdigung, wie es instinktloser nicht gedacht werden kann“,^36^ gegeißelt, wobei der anonyme Autor oder auch nur Initiator des Artikels durchaus Stark gewesen sein könnte, der damals im Zusammenhang mit der Anti‐Heisenberg‐Kampagne^37^ enge Verbindungen zur Redaktion des offiziellen Publikationsorgans der SS pflegte.

Bemerkenswert ist, dass auch Gustav Hertz, seit 1927 Physikordinarius an der TH Berlin‐Charlottenburg, von Stark aufgefordert wurde, sich an der Kundgebung der „arischen“ Nobelpreisträger zu beteiligen. Dies, obwohl er einen jüdischen Großelternteil hatte, den Vater des Physikers Heinrich Hertz, und damit nach den nationalsozialistischen Rassenkriterien als „Mischling“ galt – wie z.B. auch Otto Warburg. Allerdings hatte Stark von Gustav Hertz als Physiker eine hohe Meinung. Als das Reichserziehungsministerium diesem im Sommer 1934 die Prüfungsberechtigung entziehen wollte, setzte sich Stark für ihn ein, so dass – wohl in einer konzertierten Aktion mit der Fachschaftsvertretung der TH^38^ – beim Ministerium erreicht werden konnte, dass der Entzug der Prüfungsberechtigung für Hertz ausgesetzt wurde. In diesem Zusammenhang hatte Stark im November 1934 in einem gutachterlichen Brief an den NS‐Dozentenbund erklärt, dass

Professor Hertz in seinem Aeusseren, in seinem Auftreten und in seiner wissenschaftlichen Tätigkeit nichts Jüdisches [hat]. Es wäre eine Dummheit sondergleichen, diesem Mann deswegen die Prüfungserlaubnis zu entziehen, weil sein Grossvater Jude war. Ich bin überzeugt, dass er die ihm angetane Kränkung nicht ruhig hinnehmen, sondern sein Amt niederlegen […] würde.^39^

Gustav Hertz war zum Zeitpunkt der Aussendung des Starkschen Telegramms auf Reisen und seine Frau Ellen teilte Stark telegrafisch mit, ihren Gatten „bald möglichst vom Inhalt ihres Telegramms in Kenntnis zu setzen“.^40^ Hertz hat dann nach seiner Rückkehr in einem ausführlichen und überaus freundlichen, fast anbiedernden und mit patriotisch‐nationalistischem Pathos verfassten Brief^41^ erläutert, dass er wegen der staatlichen Diskriminierung als „Nichtarier“ den Aufruf nicht unterschreiben wolle und dafür Starks Verständnis eingefordert.

Die Reaktionen auf Starks Telegramm lassen sich insgesamt in vier Kategorien gliedern:

– Zustimmungen: Bergius, Lenard

– Bedenkenträger/hinhaltende Antworten: Bosch, Fischer, Hertz, Wieland

– Direkte Ablehnungen: Heisenberg, v. Laue, Windaus

– Polemiken/ausführliche Antworten: v. Laue, Nernst, Planck

Vorbehaltlos zustimmend hat sich nur der Heidelberger Chemiker Friedrich Bergius geäußert, der noch am selben Tag Stark telegraphisch antwortete.^42^

Zu den Unterstützern des Aufrufs muss man auch Philipp Lenard zählen, einen engen Mitstreiter in Starks Kampf gegen die moderne theoretische Physik und Urheber der sogenannten arischen „Deutschen Physik“. Dieser hatte zunächst kurz und bündig telegraphiert: „Nein ich möchte nicht mit Nobelpreisträgern unterzeichnen. Lenard“.^43^ In einem Brief vom selben Tag hatte er noch erläuternd und in Verkennung der politischen Hintergründe der Initiative geschrieben: „Ist mehr Planck oder mehr Bosch dahinter? Ich will nicht dabei sein“ und abwertend von den „Nobel‐Geschäftemachern“ gesprochen.^44^ Zwei Tage später, am 13. August, als ihm die politische Dimension der Starkschen Aktion klar geworden war, hat er seine Meinung zwar im Grundsatz revidiert, doch zeigt die Wortwahl seines Telegramms, dass ihn die Goebbels‐Starksche Initiative nach wie vor nicht voll überzeugte: „Bitte in der angefragten rohen Sache nach Gutdenken verfahren. Lenard“.^45^

Eine hinhaltende bzw. die Sache offen haltende Antwort erhielt Stark von Carl Bosch, dessen Privatsekretariat ihm zunächst mitteilte, dass wegen „Abwesenheit von Geheimrat Bosch“ das Telegramm nicht zugestellt werden könne. Auf eine nicht überlieferte briefliche Nachfrage Starks vom 22. August zu den Gründen der Nichtbeantwortung teilte dann Bosch persönlich mit, er sei leider nicht erreichbar gewesen, da er sich auf einer Erholungsreise befunden habe „ohne besonderes Ziel und mit bewußter Unterlassung der Angabe irgendeiner Adresse, um wirklich eine Erholung zu finden“.^46^

Auch die Münchener Chemiker Hans Fischer und Heinrich Wieland – Letzteren hatte Stark in der bereits erwähnten Lenard‐Stark‐Korrespondenz ebenfalls als „Münchener Chemiejude[n]“ denunziert –^47^ machten keine grundsätzlichen Bedenken gegen die geplante Kundgebung geltend, da sie „an sich durchaus unserer Einstellung [entspricht]“. Allerdings fanden sie „den gegenwärtigen Zeitpunkt zur Abgabe einer derartigen Erklärung nicht für ganz zweckmässig“, da sie im „Bewußtsein ihrer Verantwortung als Leiter grösserer Institute nicht den Eindruck erwecken wollten, als ob wir mit dieser Entwicklung einverstanden wären“.^48^

Ähnlich lavierend begründete Werner Heisenberg, warum er sich nicht der Starkschen Initiative anschließen wolle: „Obwohl ich persönlich ‚Ja‘ stimme, scheint mir politische Kundgebung von Wissenschaftlern unrichtig da auch früher niemals üblich. Unterzeichne daher nicht.“^49^ Dilatorisch war auch die Antwort des Berliner Physikochemikers Walther Nernst, der „dringend Vorberatung im kleinen Kreis“ empfahl und insbesondere auf die problematische „Verquickung von internationaler Nobelstiftung mit innerer Politik“ verwies.^50^ Beide Antworten führten zu einer harschen Reaktion Starks, deren Schärfe nur zu verstehen ist, wenn man weiß, dass Stark in beiden Erzfeinde in seinem Kampf gegen das wissenschaftliche Establishment und die moderne Physik sah. Für Nernst galt dies seit den frühen zwanziger Jahren und insbesondere, nachdem er und nicht Stark 1922 Präsident der Physikalisch‐Technischen Reichsanstalt geworden war.^51^ Heisenberg, der einer jüngeren Wissenschaftler‐Generation angehörte und nach der Nobelpreis‐Ehrung (1933) in der *physical community* als der bedeutendste und wichtigste deutsche Physiker galt, wurde von Stark regelrecht abgekanzelt. Die Verweigerung seiner Unterschrift unter dieser „nationalen Kundgebung“ erfolge mit einer „kümmerlichen Begründung“, die zeige, dass ihm „entweder das Urteil oder der nationale Instinkt“ fehle.^52^ In ähnlich scharfem Ton ist auch der Antwortbrief an Nernst gehalten, dem er vorhält, sich „mit einer schwächlichen Ausflucht“ der „nationalen Kundgebung für den Führer“ entzogen zu haben; zudem wird Nernst persönlich diskreditiert, wenn Stark ihm „einen Mangel nationaler Einstellung“ unterstellt.^53^

Dies forderte Nernst heraus, der diese Unterstellung nicht einfach hinzunehmen bereit war. Nach mehreren Entwürfen, die er direkt auf dem Brief Starks notierte (siehe **Abb.** **1** mit den entsprechenden Transkriptionen), ließ er diesem eine Antwort zukommen, die an Deutlichkeit nichts zu wünschen übrig lässt. Darin spricht er Stark das Recht ab, seine „nationale Einstellung“ zu kritisieren. Er verweist dabei auf seine Fronterfahrung und macht geltend, dass Stark, der kein aktiver Soldat gewesen war, dem nichts Gleichwertiges entgegenzusetzen habe; auch empört er sich über dessen Charakterisierung seines Handelns: Was ihm Stark als „schwächliche Ausflucht“ auslegt, sei vielmehr seine wohlbegründete Überzeugung, so Nernst, der den Brief demonstrativ „Mit deutschem Gruß“ enden lässt,^54^ um auch in der Abschiedsfloskel (statt des eigentlich korrekten „Heil Hitler“) seine patriotische Gesinnung zum Ausdruck zu bringen. Eine Antwort von Stark ist nicht überliefert.

**Abb. 1:**
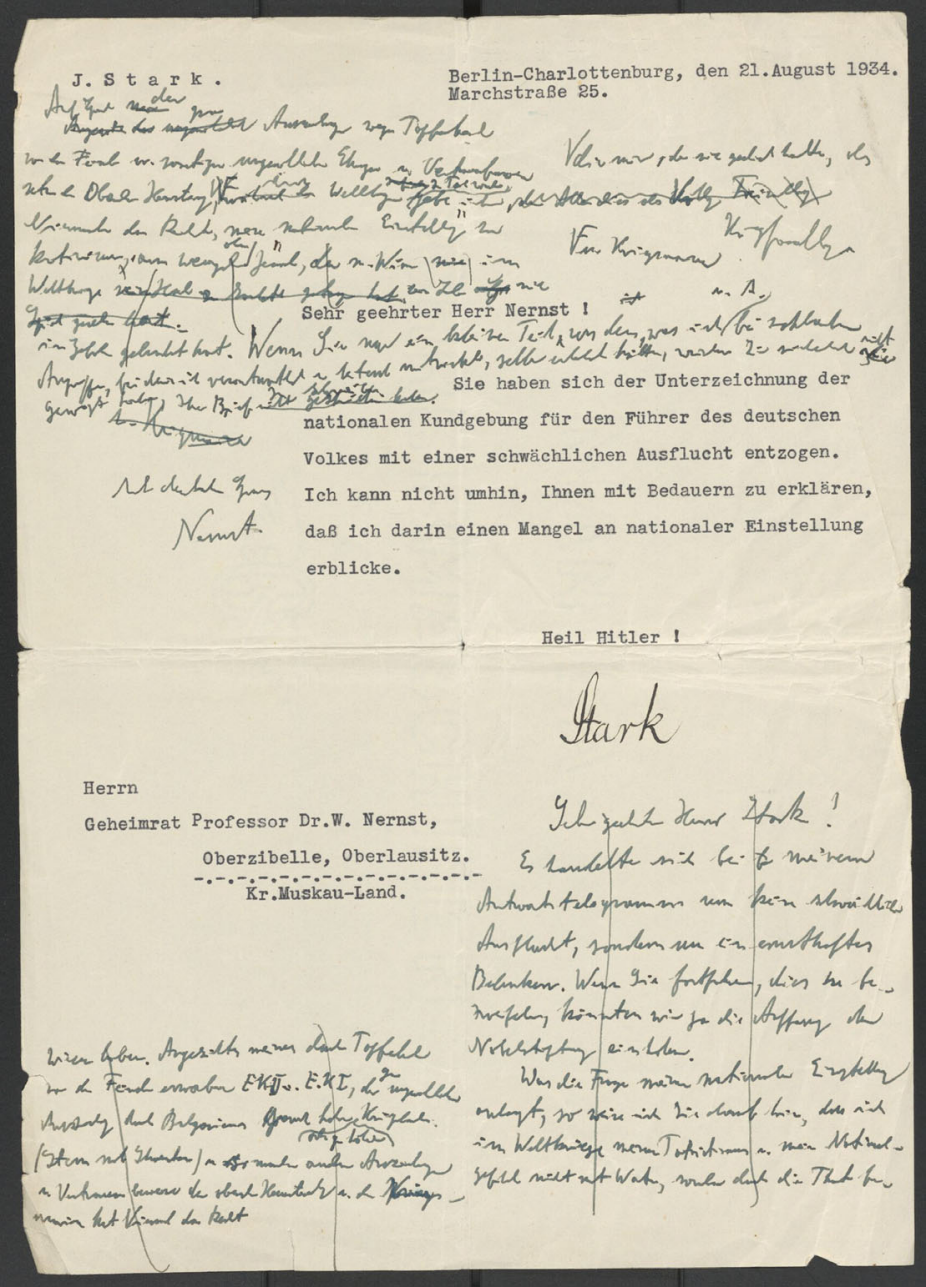
Faksimile der Entwürfe von W. Nernst für den Antwortbrief an Stark vom 1.9.1934. –Transkription links oben: „Auf Grund der grossen Auszeichnungen wegen Tapferkeit vor dem Feind u[nd] sonstigen ungewöhnlichen Ehrungen u. Vertrauensbeweisen durch die Oberste Heeresleitung u. Kriegsmarine gebe ich Niemand das Recht, meine ‚nationale Einstellung‘ zu kritisieren, am wenigsten aber jemand, der meines Wissens im Weltkriege nie im Felde geleistet hat. Wenn Sie nur einen kleinen Teil von dem was ich u[nd] A[ndere] bei zahlreichen Angriffen, bei denen ich verantwortlich u[nd] leitend mitwirkte, selber erlebt hätten, würden Sie sicherlich nicht gewagt haben, Ihren Brief zu schreiben. [darunter: „“] Mit deutschem Gruss Nernst.“ – Transkription unten: „Sehr geehrter Herr Stark! Es handelt sich bei meinem Antworttelegramm um keine schwächliche Ausflucht, sondern um ein ernsthaftes Bedenken. Wenn Sie fortfahren, dies zu bezweifeln, könnten wir ja die Auffassung der Nobelstiftung einholen. Was die Frage meiner nationalen Einstellung anlangt, so weise ich Sie darauf hin, dass ich im Weltkriege meinen Patriotismus u[nd] mein Nationalgefühl nicht mit Worten, sondern durch die That bewiesen habe. Angesichts meines durch Tapferkeit vor dem Feinde erworbene EK II u[und]. EK I, der ganz ungewollten Auszeichnungen durch Belgiens [??] hohes (oder zu hohes) Kriegs [??] (Stern mit Schwertern) u[nd] so mancher anderer Auszeichnungen u[nd] Vertrauensbeweise der obersten Heeresleitung u[nd] der Kriegsmarine hat Niemand das Recht“.

Sehr viel direkter waren die Ablehnungen des Berliner Physikers Max von Laue und des Göttinger Chemikers Adolf Windaus. Beide ließen die politischen Differenzen gegenüber Stark und damit auch zu Hitler bzw. dem Nationalsozialismus unmissverständlich erkennen, indem sie kurz und bündig mitteilten: „Ich bin gegen politische Kundgebung von Gelehrten“ (Laue)^55^ bzw. „Ich unterzeichne keine Aufrufe für Hitler“ (Windaus).^56^

Insbesondere durch von Laues Reaktion fühlte sich Stark herausgefordert, dem er in seinem Antwortbrief Inkonsistenz des eigenen Verhaltens vorwarf. Denn dieser habe doch unlängst auf der Würzburger Physiker‐Tagung im September 1933 – dort war Starks Versuch, die Präsidentschaft der Deutschen Physikalischen Gesellschaft zu übernehmen, nicht zuletzt an von Laues couragiertem Widerspruch gescheitert^57^ – „sich nicht gescheut, zugunsten von Albert Einstein diesen Landesverräters und Beschimpfers der nationalsozialistischen Regierung, unter dem Beifall der anwesenden Juden und Juden‐Genossen eine öffentliche Kundgebung zu veranstalten, die sich letzten Endes gegen die nationalsozialistische Regierung richtete“. Wegen „dieses unterschiedliche[n] Verhalten[s]“ meinte er, von Laue sein „stärkstes Bedauern“ aussprechen zu müssen.^58^

Von besonderem Interesse ist die Korrespondenz, die sich anknüpfend an Starks Telegramm mit Max Planck entwickelte. Mehr noch als von Laue oder Nernst war Planck *der* Intimfeind von Stark, was weniger persönlichen Animositäten oder physikalischen Kontroversen geschuldet war. Vielmehr sah Stark in Planck den „Hauptförderer Einsteins und des jüdischen Einflusses in der Wissenschaft unter dem schwarz‐roten System“,^59^ was er seit den zwanziger Jahren mit wachsendem Furor zu bekämpfen suchte.^60^ Zahlreich sind die Sottisen zu Planck in der Korrespondenz zwischen Stark und Philipp Lenard – so wird er beispielsweise als „arischer Juden‐Don‐Quichote“ karikiert,^61^ und immer wieder polemisierte Stark mit scharfen und aggressiven Formulierungen gegen Planck und diffamierte ihn wahlweise als „Judengenossen“, „Judengünstling“ oder „Judengönner“.^62^ Scharf fiel deshalb auch seine Antwort auf Plancks telegraphische Erklärung aus, dass er sich „grundsätzlich nicht persönlich an öffentlichen Kundgebungen politischen Charakters“ beteilige:^63^ Damit habe Planck als „einer der führenden Wissenschaftler“ nicht erkannt, dass diese

Kundgebung für den Führer des deutschen Volkes nicht eine politische Kundgebung früheren Stils sein [sollte], sondern ein Teil des großen nationalen Bekenntnisses des ganzen deutschen Volkes vor der ganzen Welt. Es ist bedauerlich, daß Sie als einer der führenden Wissenschaftler diesen klaren Tatbestand nicht erkannt [haben]. Es ist dies um so bedauerlicher, als Sie an der Spitze der Kaiser‐Wilhelm‐Gesellschaft stehen und den Führer persönlich kennen.^64^

Allerdings war gerade die KWG in den ersten Jahren des Dritten Reiches bei den Auseinandersetzungen um die nationalsozialistische Neuordnung der deutschen Forschung starken Angriffen ausgesetzt,^65^ die auf ihre Nivellierung, ja Auflösung zielten. Als der damalige „starke Mann der deutschen Wissenschaftspolitik“^66^ spielte Johannes Stark dabei eine zentrale Rolle, führte er doch die Fronde gegen die KWG an.

Stark hat Plancks Ablehnung, die er rückblickend als „eine Kundgebung politischen Charakters, wenn auch im kleinen Kreise“ charakterisierte,^67^ Jahre später, in einem unpublizierten Manuskript aus den Jahren 1937/38 („Planck als Politiker“^68^), nochmals thematisiert und mit gänzlich anderen Kontexten in Beziehung gesetzt.^69^ Es sind nun nicht Plancks Ämter, sondern dessen öffentlichen Vorträge^70^, die er verstärkt ab Mitte der dreißiger Jahre „in einer Reihe von Städten unter großem Zulauf gehalten [hatte]“.^71^ Speziell geht es um Plancks Vortrag zum Verhältnis von „Religion und Naturwissenschaft“ aus dem Jahr 1937,^72^ den Stark – sicher zu Recht^73^ – als gegen den Nationalsozialismus gerichtet charakterisiert, was Planck in anderer Weise ebenfalls zum „Politiker“ mache.^74^

In seinem Antwortschreiben^75^ ließ Planck die aktuellen Konflikte um die Kaiser‐Wilhelm‐Gesellschaft gänzlich unerwähnt. Sein ablehnendes Verhalten gegenüber der geplanten Kundgebung begründete er mit einer prägenden Erfahrung aus dem Ersten Weltkrieg: dem auch von ihm unterschriebenen *Aufruf an die Kulturwelt* vom Herbst 1914, der den deutschen Überfall auf das neutrale Belgien zu rechtfertigen suchte und den deutschen Militarismus als Schutzpatron deutscher Kultur hochstilisierte: „Ohne den deutschen Militarismus wäre die deutsche Kultur längst von Erdboden getilgt […]. Deutsches Heer und deutsches Volk sind eins.“^76^ Dieser „Aufruf der 93“, dem – in den Worten Plancks – „echt patriotische Gesinnung eingegeben war, [der] aber sein Ziel völlig verfehlte“,^77^ hatte dem Ansehen der deutschen Wissenschaft nicht nur bei den Kriegsgegnern, sondern auch im neutralen Ausland massiv geschadet^78^ – nicht zuletzt Planck persönlich, obwohl dieser noch während des Ersten Weltkriegs (1916) zu einer differenzierten Sichtweise fand.^79^ Angesichts der verheerenden Wirkung des Aufrufs hatte Planck „ein Gelübde getan, [sich] nie wieder an einer derartigen mir vorgelegten öffentlichen Kundgebung zu beteiligen“.^80^

Planck hat im Übrigen als einziger der von Stark angeschriebenen Nobelpreisträger seine Antwort mit einem ausdrücklichen Bekenntnis zu Hitler angereichert: „unserem Führer, den ich persönlich hoch verehre“.^81^ Anders als Stark war Planck jedoch alles andere als ein begeisterter Anhänger des Nationalsozialismus oder gar Hitlers. Deshalb lässt sich diese Formulierung auch als Ausdruck seiner Strategie gegenüber den neuen Machthabern deuten. In den ersten Jahren der NS‐Diktatur hatte er die Illusion, dass es durch geschicktes Taktieren gelingen könnte, die deutsche Wissenschaft auch unter der nationalsozialistischen Regierung vor größerem Schaden zu bewahren. Dabei war sein Handeln „darauf gerichtet, jeden öffentlichen Protest oder gar eine dezidierte Frontstellung gegenüber den Nationalsozialisten zu vermeiden und so einen vertrauensvollen Aufbau von Beziehungen zu den neuen Machthabern nicht zu gefährden“.^82^ Plancks Elogen „für unseren Führer“^83^ dürften Stark selbst kaum beeindruckt haben, war doch der Dissens zwischen beiden unüberbrückbar groß, was nicht zuletzt in dem oben erwähnten unveröffentlichten Manuskript zum Ausdruck kommt. Allerdings musste er damit rechnen, dass Stark seinen Brief an übergeordnete Stellen in Partei und Regierung weitergeben würde. Damit war ein verbindlicher Ton angesagt, der sich allerdings auch als Kooperationsbereitschaft an die neuen Machthaber lesen läßt.

Die Weitergabe der Korrespondenz ist dann auch tatsächlich erfolgt, denn vier Tage nach dem Referendum, das erwartungsgemäß eine überwältigende Mehrheit für Adolf Hitler erbrachte, wandte sich Stark noch persönlich an Propagandaminister Josef Goebbels, um über den Misserfolg seiner Bemühungen zu berichten und ihm gleichzeitig auch denunziatorisch die „Antworten meiner Kollegen auf mein Telegramm, sowie meine Antwortschreiben“ zur Kenntnis zu bringen.^84^

Mit den Erklärungen der elf von Stark angeschriebenen Nobelpreisträger waren die Frontlinien klar gezogen. Da nur zwei von ihnen bereit waren, den Starkschen Aufruf zu unterzeichnen, musste das Vorhaben zu den Akten gelegt werden. Stark stand als Verlierer da und hatte sich blamiert. Wie Beyerchen in diesem Zusammenhang ausführt,^85^ machte die Niederlage deutlich, dass Stark, der damals im Zenit seiner wissenschaftspolitischen Macht und Einflussmöglichkeiten stand, kaum über Rückhalt in der *scientific community* verfügte. Darüber herrschte im Übrigen nicht nur unter Starks Fachkollegen weitgehend Konsens, es war auch die Meinung von Teilen der Ministerial‐Bürokratie. In einem Brief von Wissenschaftsminister Bernhard Rust an Hitler aus dem Jahre 1936 findet sich die Feststellung, „daß er [Stark] von vielen namhaften führenden Männern und hohen Staatsstellen abgelehnt wird, daß die Zusammenarbeit auf größte Schwierigkeiten stoßen müßte“.^86^ Starks undiplomatisches und autokratisches Verhalten hatte ihn nicht zufällig Spitznamen wie „Johannes Robustus“^87^ oder „Giovanni Fortissimo“^88^ eingebracht, was seine Reaktionen auf die Antworten der kontaktierten Nobelpreisträger geradezu typisch machen.

Damit war der Schlussstrich unter eine Aktion gezogen, die bislang in den wissenschaftshistorischen Darstellungen zum Dritten Reich kaum Beachtung gefunden hat. Erwähnung findet Starks Initiative in Alan Beyerchens immer noch grundlegender Monographie über die Physiker im Dritten Reich;^89^ fast beiläufig ist die Erwähnung in Fritz Sterns Berliner Vortrag von 1997 zum 50. Todestag von Planck.^90^ Etwas ausführlicher gehen Katharina Zeitz in ihrer Dissertation zu Max von Laue,^91^ John Heilbron in seiner Planck‐Biographie^92^ und auch die Heisenberg‐Biographie von David Cassidy^93^ darauf ein. Allerdings waren bisher die meisten Antworten auf Starks Telegramm sowie die sich daraus ergebenden Korrespondenzen (wenn überhaupt) nur in Teilen bekannt und wurden erst jüngst von den Autoren im Familiennachlass von Johannes Stark aufgefunden.

## Anhang: Dokumente^94^

**1.**

**Telegramm von Johannes Stark an Nobelpreisträger, ohne Datum [11.8.1934]**

Deutsches Museum, München, Archiv: HS 1946‐07/1 Akte (1)^95^

Umgehend Antwort erbeten, ob Sie zusammen mit anderen Nobelpreisträgern folgende Kundgebung unterzeichnen wollen: In Adolf Hitler verehren und bewundern wir deutsche Naturforscher den Retter und Führer des deutschen Volkes. Unter seinem Schutz und seiner Förderung wird unsere wissenschaftliche Arbeit dem deutschen Volke dienen und das deutsche Ansehen in der Welt mehren.

Stark, Notgemeinschaft der deutschen Wissenschaft, Schloß.

**2.**

**Telegramm von Friedrich Bergius an Stark, 11.8.1934**

Familienarchiv Stark

Kundgebung entspricht ganz meiner eigenen Überzeugung. Ich unterstütze deshalb mit Freuden. Bergius

**3.**

**Telegramm von Carl Bosch an Stark, 11.8.1934**

Familienarchiv Stark

Wir bestätigen in Abwesenheit von Geheimrat Bosch Ihr freundliches Telegramm, das wir am Montag an Geheimrat Bosch weiterleiten werden, da uns erst bis dahin die neue Adresse bekanntgegeben wird. Privatsekretariat Bosch.

**3A.**

**Brief von Carl Bosch an Stark, Heidelberg, 28.8.1934**

Familienarchiv Stark [Original, Schreibmaschine]

Dr. C. Bosch             Heidelberg, den 28. August 1934.

Herrn

Präsidenten Professor Dr. Stark

Berlin‐Charlottenburg,

Marchstrasse 25.

Sehr verehrter Herr Präsident!

Ich fand bei der Rückkehr von einer Erholungsreise Ihren Brief vom 22. d.M. hier vor. Was Ihre Anfrage nach den Gründen für die Nichtbeantwortung Ihres Telegramms vom 11. d.M. anbetrifft, so teile ich Ihnen mit, daß sie darin zu suchen sind, daß ich eine Erholungsreise angetreten hatte ohne besonderes Ziel und mit bewußter Unterlassung der Angabe irgendeiner Adresse, um wirklich eine Erholung zu finden. Als Ihr Telegramm in meine Hände kam, war es für eine Antwort zu spät. Ich gebe zu, daß es ein Fehler von mir war, Ihnen nicht trotzdem geantwortet zu haben.

Mit vorzüglicher Hochachtung

C. Bosch

**4.**

**Telegramm von Werner Heisenberg an Stark. Menzenschwand, 15.8.1934**

Familienarchiv Stark

Obwohl ich persönlich „Ja“ stimme scheint mir politische Kundgebung von Wissenschaftlern unrichtig da auch früher niemals üblich. Unterzeichne daher nicht. Heisenberg

**4A.**

**Antwortschreiben Stark an W. Heisenberg, Bln.‐Charlbg., 21.8.1934**

Familienarchiv Stark

Sehr geehrter Herr Heisenberg!

Sie haben die Unterzeichnung der nationalen Kundgebung für den Führer des deutschen Volkes abgelehnt mit der kümmerlichen Begründung, es sei eine politische Kundgebung. – damit haben Sie gezeigt, daß Ihnen entweder das Urteil oder der nationale Instinkt fehlt. Heil Hitler!

gez. Stark.

**5.**

**Telegramm von Ellen Hertz an Stark, 11.8.1934**

Familienarchiv Stark

Mein Mann ist zur Zeit verreist mit wechselnder Adresse. Werde versuchen ihn bald möglichst vom Inhalt Ihres Telegramms in Kenntnis zu setzen. Frau Hertz

**5A.**

**Brief von Gustav Hertz an Stark, Berlin, 15.8.1934**

Familienarchiv Stark [Original, Schreibmaschine]

Prof. Dr. G. Hertz                Berlin‐Dahlem, 15.8.34.

                    Fabeckstrasse 11

Hochverehrter Herr Präsident,

infolge eines unglücklichen Zufalls hat mich Ihr Telegramm mit der Aufforderung zur Unterzeichnung einer Kundgebung erst bei meiner Rückkehr von einer kurzen Reise erreicht, so dass es mir leider nicht möglich war, Ihnen rechtzeitig zu antworten.

Als ein Mann, der stets nur deutsch gefühlt hat, dessen ehrliches Bestreben es stets gewesen ist, deutsch zu handeln, und dem niemand vorwerfen kann, dass er zu irgend einer Zeit im Kriege oder im Frieden Mangel an nationaler Gesinnung gezeigt habe, gelte ich noch heute, meines einen nicht arischen Grossvaters wegen, als Nichtarier. Wenn ich auch in der Ausübung meiner wissenschaftlichen Tätigkeit nicht behindert bin, so bin ich doch von allen Ehrenämtern ausgeschlossen, ja ich werde sogar als unwürdig betrachtet, Mitglied irgend einer Organisation zu werden. Ich hoffe zuversichtlich, dass in nicht zu ferner Zeit ein Weg gefunden werden wird, um diesen für mich auf die Dauer unerträglichen Zustand zu beenden und Männer meiner Art wieder als vollberechtigte Mitglieder in die Deutsche Volksgemeinschaft aufzunehmen. Bis dahin gibt es für mich in meiner jetzigen Lage nur den einen Weg, mich jeder Betätigung in der Oeffentlichkeit zu enthalten und mich darauf zu beschränken, durch wissenschaftliche Arbeit und Ausbildung junger deutscher Physiker meine Pflicht zu tun. Hierdurch werde ich gleichzeitig, so hoffe ich, dem Ansehen der deutschen Wissenschaft in der Welt dienen können.

In der Hoffnung, dass Sie für diesen meinen Standpunkt Verständnis haben werden, bin ich mit deutschem Gruss

Ihr sehr ergebener

G. Hertz.

**6.**

**Telegramm von Max von Laue an Stark, 13.8.1934**

Familienarchiv Stark

Ich bin gegen jede politische Kundgebung von Gelehrten. Laue

**6A.**

**Brief von Stark an Max von Laue, 21.8.1934**

Archiv der Max‐Planck‐Gesellschaft, Berlin: MPGA IX. Abt., Rep. 4 1933–1934. Veröffentlicht in Hoffmann u. Walker 2007, S. 547.

Sehr geehrter Herr v. Laue!

Sie haben die von mir angeregte Kundgebung mit der Unterstellung abgelehnt, es sei eine politische Kundgebung von Gelehrten. Diese Unterstellung ist falsch. Sie sollte in Wirklichkeit ein Teil des großen nationalen Bekenntnisses des deutschen Volkes zu seinem Führer Adolf Hitler vor aller Welt sein. Jetzt haben Sie eine öffentliche Kundgebung für Adolf Hitler abgelehnt, dagegen haben Sie auf der Würzburger Physiker‐Tagung sich nicht gescheut, zugunsten von Albert Einstein, diesen Landesverräters und Beschimpfers der nationalsozialistischen Regierung, unter dem Beifall der anwesenden Juden und Juden‐Genossen eine öffentliche Kundgebung zu veranstalten, die sich letzten Endes gegen die nationalsozialistische Regierung richtete. Ich kann nicht umhin, Ihnen über dieses unterschiedliche Verhalten mein stärkstes Bedauern auszudrücken.

Heil Hitler!

Stark

**7.**

**Telegramm von Philipp Lenard an Stark, 11.8.1934**

Familienarchiv Stark

Nein ich möchte nicht mit Nobelpreisträgern unterzeichnen. Lenard

**7A.**

**Brief von Philipp Lenard an Stark, Heidelberg, 11.8.1934**

Staatsbibliothek zu Berlin – Preußischer Kulturbesitz, Nachlass Johannes Stark [Original, handschriftlich]

Lieber Stark!

Soeben habe ich Ihr Telegramm wegen den Nobel‐Geschäftemachern beantwortet. Ist mehr Planck oder mehr Bosch dahinter? Ich will nicht dabei sein. [. . .]

**7B.**

**Telegramm von Philipp Lenard an Stark, 13.8.1934**

Familienarchiv Stark

Bitte in der angefragten rohen Sache nach Gutdenken verfahren. Lenard

**8.**

**Telegramm von Walther Nernst an Stark, 13.8.1934**

Familienarchiv Stark

Hege Bedenken gegen Verquickung von internationaler Nobelstiftung mit innerer Politik. Empfehle dringend Vorberatung im kleinen Kreise. Nernst

**8A.**

**Brief von Stark an Walther Nernst. Berlin, 21. August 1934**

Deutsches Museum, München, Archiv: HS 1946‐07/1 Akte [Original, Schreibmaschine]

J. Stark          Berlin‐Charlottenburg, den 21. August 1934

             Marchstraße 25.

Sehr geehrter Herr Nernst!

Sie haben sich der Unterzeichnung der nationalen Kundgebung für den Führer des deutschen Volkes mit einer schwächlichen Ausflucht entzogen. Ich kann nicht umhin, Ihnen mit Bedauern zu erklären, daß ich darin einen Mangel nationaler Einstellung erblicke.

Heil Hitler! Stark

**8B.**

**Brief von Walther Nernst an Stark, 1.9.1934**

Familienarchiv Stark [Original, handschriftlich]^96^

Betr. Ihr Schreiben vom 21.8.34.     Zibelle (O.L.) bei Muskau, 1.9.34.

Sehr geehrter Herr Stark!

Auf Grund der grossen Auszeichnungen wegen Tapferkeit vor dem Feinde u. sonstigen ungewöhnlichen Ehrungen u. Vertrauensbeweisen, die mir, der ich nie gedient hatte, als Kriegsfreiwilliger seitens der Obersten Heeresleitung u. Kriegsmarine während des Weltkrieges zu Teil geworden sind, gebe ich Niemandem das Recht, meine „nationale Einstellung“ zu kritisieren. Wenn Sie nur einen kleinen Teil von dem, was ich u. A. bei zahlreichen Angriffen, bei denen ich in vorderster Linie verantwortlich u. leitend mitgewirkt habe, selber erlebt hätten, würden Sie sicherlich nie gewagt haben, Ihren Brief zu schreiben. Im Uebrigen ist das, was Sie „schwächliche Ausflucht“ nennen, meine wohlbegründete Ueberzeugung.

Mit deutschem Gruss

Nernst.

**9.**

**Telegramm von Max Planck an Stark, 12.8.1934**

Familienarchiv Stark

Beteilige mich grundsätzlich nicht persönlich an öffentlichen Kundgebungen politischen Charakters. Bedaure sehr in vorliegendem Falle keine Ausnahme machen zu können. Planck

**9A.**

**Brief von Stark an Max Planck, 20.8.1934**

Familienarchiv Stark [Durchschlag, Schreibmaschine]

J. Stark.        Charlottenburg, Marchstraße 25, 20. August 1934.

Sehr geehrter Herr Planck!

Sie haben die Unterzeichnung der von mir angeregten Kundgebung für den Führer mit der Begründung abgelehnt, es handele sich um eine politische Kundgebung. Diese Begründung ist falsch. Die Kundgebung für den Führer des deutscher Volkes sollte nicht eine politische Kundgebung früheren Stiles sein, sondern ein Teil des großen nationalen Bekenntnisses des ganzen deutschen Volkes vor der ganzen Welt. Es ist bedauerlich, daß Sie als einer der führenden Wissenschafter diesen klaren Tatbestand nicht erkannt und sich nicht öffentlich zum Führer des deutschen Volkes bekannt haben. Es ist dies um so bedauerlicher, als Sie an der Spitze der Kaiser‐Wilhelm‐Gesellschaft stehen und den Führer persönlich kennen.

Heil Hitler!

gez. Stark

Herrn

Geheimen Regierungsrat Professor

Dr. M. Planck,

Berlin‐Grunewald.

Wangenheimstraße 21.

**9B.**

**Brief von Max Planck an Stark, 27.8.1934**

Familienarchiv Stark [Original, handschriftlich]

z.Z. Schmieden bei Villabassa (Bolzano)

27.8.34.

Sehr geehrter Herr Kollege!

Es tut mir sehr leid, aus Ihrem Schreiben vom 21. d. M. zu erfahren, daß Sie mein Verhalten zu der von Ihnen angeregten Kundgebung mißbilligen.

Ich kann dazu nur sagen, daß ich bei einer früheren Veranlassung, nach dem „Aufruf der 93“ vom Jahre 1914, der gewiß von echt patriotischer Gesinnung eingegeben war, aber sein Ziel völlig verfehlte, ein Gelübde getan habe, mich nie wieder an einer derartigen mir vorgelegten öffentlichen Kundgebung zu beteiligen, da sie doch stets von gewissen Leuten mißverstanden wird. Im Uebrigen liegt mir daran, zu betonen, daß ich keine passende Gelegenheit versäume, um mündlich oder schriftlich, privatim oder öffentlich, mich zu unserem Führer, den ich persönlich hoch verehre, ausdrücklich zu bekennen, das letzte Mal öffentlich bei der diesjährigen Tagung der Kaiser‐Wilhelm‐Gesellschaft, und daß ich während der kurzen Zeit, die mich noch an mein Amt in der K.W.G. binden wird, selbstverständlich bei dieser Gewohnheit zu bleiben gedenke.

Mit kollegialem Gruß und Heil Hitler Ihr

Planck

**10.**

**Brief von Heinrich Wieland und Hans Fischer an Stark, 12.8.1934**

Familienarchiv Stark [Original, handschriftlich]

Ambach a. Starnberger See

12. August 1934

Sehr geehrter Herr Kollege.

Der Inhalt der Kundgebung, die Sie gemeinsam mit andern Nobelpreisträgern anregen, entspricht an sich durchaus unsrer Einstellung. Jedoch halten wir den gegenwärtigen Zeitpunkt zur Abgabe einer derartigen Erklärung nicht für ganz zweckmässig.

Wir sehen in der vor sich gehenden Entwicklung unsres Hochschullebens, vor allem in der grundlegenden Frage der Besetzung der Lehrstühle, ernste Gefahren für den Fortschritt der deutschen Wissenschaft und möchten, im Bewusstsein unsrer Verantwortung als Leiter grösserer Institute, nicht den Eindruck erwecken, als ob wir mit dieser Entwicklung einverstanden wären.

Mit deutschem Gruss

Ihre sehr ergebenen

H. Wieland H. Fischer.

**10A.**

**Telegramm von Hans Fischer an Stark, 14.8.1934**

Familienarchiv Stark

Sonntag brieflich mit Wieland beantwortet. Fischer

**11.**

**Adolf Windaus an Stark**

Archiv der Universität Göttingen: Cod. Ms. A. Windaus A60 [Handschriftlicher Entwurf]

Ich unterzeichne keine Aufrufe für Hitler. Windaus

**11A.**

**Brief von Stark an Adolf Windaus, 21.8.1934**

Archiv der Universität Göttingen: Cod. Ms. A. Windaus A60 [Original, Schreibmaschine]

J. Stark

Berlin‐Charlottenburg den 21. Aug. 1934.

Marchstraße 25.

Sehr geehrter Herr Windaus!

Sie haben es fertiggebracht zu erklären, daß Sie die Kundgebung für Adolf Hitler nicht unterzeichnen. Ich bin empört über Ihre in schroffer Form gehaltene Erklärung. Sie ist nach meiner Meinung unvereinbar mit den Pflichten eines Beamten im nationalsozialistischen Reich.

Heil Hitler!

Stark

**12.**

**Brief von Stark an Joseph Goebbels, 23.8.1934**

Bundesarchiv Berlin‐Lichterfelde: R 15.19, Bd. 65, Bl. 276 [Durchschlag, Schreibmaschine]

Prof. Dr. J. Stark

Der Präsident der Physikalisch‐Technischen Reichsanstalt

Bln.‐Charlottenburg, Marchstr. 25. d. 23.8.34.

Sehr verehrter Herr Minister,

Auf Veranlassung Ihres Herrn Oberregierungsrates Dr. Ziegler habe ich gerne den Versuch gemacht, eine öffentliche Kundgebung der 12 arischen deutschen Nobelpreisträger für Adolf Hitler herbeizuführen. Wie Sie bereits wissen, ist dieser Versuch gescheitert.

Es dürfte Sie interessieren, aus den Anlagen die Antworten meiner Kollegen auf mein Telegramm, sowie meine Antwortschreiben darauf kennenzulernen.

In grösster Verehrung

Heil Hitler

Ihr sehr ergebener

An den

Herrn Reichsminister Dr. J. Goebbels,

Reichspropagandaministerium,

Berlin W 8

Wilhelmplatz 8/9.

## Conflict of Interest

The authors declare no conflict of interest.

## Data Availability

The data that support the findings of this study are available from the corresponding author upon reasonable request.

## Footnotes

1 Zur Biographie Johannes Starks siehe: Stark 1987; Beyerchen 1980, S. 146–171 (engl. Original: Beyerchen 1977); Hoffmann 1982; Walker 1995, S. 5–40.

2
Hermann 1969, S. 86–101.

3 Stark an Max Iklé, 22.2.1915, Staatsbibliothek zu Berlin – Preußischer Kulturbesitz, Handschriftenabteilung, Nachlass Johannes Stark.

4 Walker 2025, S. 310–312.

5
Stark 1937.

6 Stark an Philipp Lenard, Eppenstadt, 3.2.1933, zit. in: Kleinert 1980, S. 35.

7 Siehe Cahan 1992, S. 238–245.

8
Laue 1947, S. 272.

9
Flachowsky 2008, S. 163–200.

10
Kern 1994, S. 258.

11
Hoffmann 2007.

12
Zur Biographie P. v. Hindenburgs und speziell zu seinem Tod und Erbe siehe Pyta 2007, S. 855–877.

13
Jung 1995, S. 62.

14 Ebd., S. 133.

15
„Bericht aus Bayern“ 1934.

16
„Deutsche Hochschullehrer“ 1933.

17 Erklärung von Vertretern der deutschen Wissenschaft zum Volksentscheid vom 19.8.1934, Deutsches Museum München, Archiv, Splitternachlaß W. Nernst HS 1946‐07/1.

18
Friedman 2001, S. 251–252.

19 Anhang: Dokument Nr. 12.

20
Klee 2016, S. 694.

21 Stark an Wilhelm Ziegler, Berlin im Oktober 1937, Bundesarchiv Berlin R 15.19, Nr. 71, Bl. 62.

22 Ebd., Bl. 62R

23 Anhang: Dokument Nr. 1.

24 Stark an Philipp Lenard, Eppenstatt, 9.2.1933, Staatsbibliothek zu Berlin – Preußischer Kulturbesitz, Handschriften‐Abteilung, Nachlass Johannes Stark.

25
Nachmansohn und Schmid 1988, S. 200–235; Deichmann 2001, S. 450–467.

26
Werner 1991, S. 281–317.

27 Ebd., S. 310.

28 Brescius 1977, S. 220–298.

29
Mendelssohn 1996, Bd. 3, S. 180–183.

30 Albert Einstein Archives, Hebrew University Jerusalem.

31 James Franck Papers, Regenstein Library University of Chicago.

32 Nachlassverzeichnis Richard Willstätter, ETH Zürich, Zentralbibliothek. Siehe auch: Willstätter 1949.

33
Clary 2022, S. 46–112 sowie Hoffmann 1984, S. 59–53.

34
Stark 1936, S. 3.

35 Stark an Gunter d’Alquen, Berlin, 6.9.1937, Staatsbibliothek zu Berlin – Preußischer Kulturbesitz, Handschriften‐Abteilung, Nachlass Johannes Stark.

36
„Kehrseite der Medaille“ 1937; auch abgedruckt in Hoffmann und Walker 2007, S. 587.

37 Siehe Beyerchen 1980, S. 215–220.

38
Walcher 1987, S. 624. Siehe auch Walchers Gedächtnisprotokoll vom Treffen mit Stark am 11. November 1934 (Kopie in Sammlung D. Hoffmann, Berlin) sowie Schröder 1993, S. 332.

39 Stark an Reichsleitung NSDAP, Deutsche Dozentenschaft, Berlin, 8.11.1934, Bundesarchiv Berlin, R 15.19, Nr. 65, Bl. 171.

40 Anhang: Dokument Nr. 5.

41 Anhang: Dokument Nr. 5A.

42 Anhang: Dokument Nr. 2

43 Anhang: Dokument Nr. 7.

44 Anhang: Dokument Nr. 7A.

45 Anhang: Dokument Nr. 7B.

46 Anhang: Dokument Nr. 3A.

47 Stark an Philipp Lenard, Eppenstatt, 9.2.1933, Staatsbibliothek zu Berlin – Preußischer Kulturbesitz, Handschriften‐Abteilung, Nachlass Johannes Stark.

48 Anhang: Dokument Nr. 10.

49 Anhang: Dokument Nr. 4.

50 Anhang: Dokument Nr. 8.

51
Beyerchen 1980, S. 157.

52 Anhang: Dokument Nr. 4A.

53 Anhang: Dokument Nr. 8A.

54 Anhang: Dokument Nr. 8B.

55 Anhang: Dokument Nr. 6.

56 Anhang: Dokument Nr. 11.

57
Beyler 2007, S. 73f.

58 Anhang: Dokument Nr. 6A.

59 Stark an Wilhelm Frick, Berlin, 21.2.1935, Bundesarchiv Berlin R 15.01, Nr. 5446, Bl. 115.

60
Stark 1922.

61 Philipp Lenard an Stark, Heidelberg, 25.2.1935, Staatsbibliothek zu Berlin – Preußischer Kulturbesitz, Handschriften‐Abteilung, Nachlass Johannes Stark.

62 Philipp Lenard an Stark, Heidelberg, 27.2.1935, Staatsbibliothek zu Berlin – Preußischer Kulturbesitz, Handschriften‐Abteilung, Nachlass Johannes Stark.

63 Anhang: Dokument Nr. 9.

64 Anhang: Dokument Nr. 8A.

65
Hachtmann 2007, Bd. 1, S. 465–471.

66 Ebd., S. 465. Siehe auch: Hoffmann und Walker 2007, S. 530–561.

67 Johannes Stark, „Max Planck als Politiker“, unveröffentlichtes Manuskript, o.D. (wahrscheinlich 1937/38), Familienarchiv Stark, Bl. 4.

68 Ebd.

69 Siehe Hoffmann (in Vorbereitung).

70
Planck 1949a, 1949b.

71 Johannes Stark, „Max Planck als Politiker“, unveröffentlichtes Manuskript, o.D. (wahrscheinlich 1937/38) Familienarchiv Stark, Bl. 1. Siehe auch: Hoffmann 2008, S. 101–102.

72 Planck 1937.

73
Hoffmann 2008, S. 102.

74 Johannes Stark, „Max Planck als Politiker“, unveröffentlichtes Manuskript, o.D. (wahrscheinlich 1937/38), Familienarchiv Stark, Bl. 1.

75 Anhang: Dokument Nr. 9B.

76
Ungern‐Sternberg und Ungern‐Sternberg 2014, S. 210.

77 Anhang: Dokument Nr. 9B.

78 Siehe Ungern‐Sternberg und Ungern‐Sternberg 2014, S. 276–277.

79 Ebd.

80 Anhang: Dokument Nr. 9B.

81 Ebd.

82
Hoffmann 2008, S. 88. Siehe auch: Albrecht 1993; Hachtmann 2008; Stern 2000.

83 Anhang: Dokument Nr. 9B.

84 Anhang: Dokument Nr. 12.

85
Beyerchen 1980, S. 165.

86 Bernhard Rust (REM) an Adolf Hitler, Berlin, 2.2.1936, zit. nach: Kohl 2002, S. 133.

87 Max v. Laue an Gustav Mie, Berlin, 22.11.1934, Archiv der Max‐Planck‐Gesellschaft, Rep. 50, Ordner 1369, Bl. 2–3. Zitat auch in: Hentschel 1996, S. 90. Frau L. Stolz (Halle) half, die Quelle des Zitats zu identifizieren.

88 Arnold Sommerfeld an Albert Einstein, München, 30.12.1937, in: Sommerfeld 2004, S. 445.

89
Beyerchen 1980, S. 165–166.

90
Stern 2000, S. 57.

91
Zeitz 2006, S. 46–47.

92
Heilbron 2006, S. 242–244 (zuerst: Heilbron 1986).

93
Cassidy 1992, S. 413.

94 Der Text der Telegramme wurde vereinheitlicht, postalische Besonderheiten wie durchgehende Groß‐ oder Kleinschreibung und Vermerke wie eingestreutes STOP werden in der Transkription nicht berücksichtigt. Die Briefe werden originalgetreu wiedergegeben, d.h. unter Beibehaltung von Orthographie und Interpunktion der Vorlagen.

95 Eine leicht abweichende Fassung des Telegramms, die bei der Nachsendung des Telegramms an Windaus, der in Baden Baden zur Kur war, entstanden ist, ist im Nachlass von Adolf Windaus im Archiv der Universität Göttingen überliefert.

96 Zu diesem Brief gibt es noch zwei handschriftliche Entwürfe, notiert auf Starks Brief an Nernst vom 21. August (Dokument Nr. 8), siehe Abb. 1.
